# You Are Not Working for Me; I Am Working with You

**DOI:** 10.1371/journal.pcbi.1004387

**Published:** 2015-09-24

**Authors:** Florian Markowetz

**Affiliations:** University of Cambridge, Cancer Research UK Cambridge Institute, Cambridge, United Kingdom; EMBL Heidelberg, GERMANY

Since 2009, I have led a cancer research group at the University of Cambridge; the current group includes ten scientists (five postdocs, five PhD students). In the following, I will share with you some of the lessons I learned over the years and some of the leadership strategies that work well for me. Key topics will be the integration of new lab members and the communication in the lab (in particular, how to make expectations explicit).

## How the Lab Started

One of the papers that impressed me most as a PhD student was Eran Segal’s paper on module networks in yeast [1]. When I prepared to start my own lab, at the end of my postdoc in 2008, I realised there had been almost no follow-up, and certainly nothing in cancer research. What an opportunity, I thought! So I wrote up a series of projects, which could easily have kept two postdocs busy for three years, on how to extend module networks and use them for data integration in cancer genomics. It was a great plan. Then I went to Intelligent Systems in Molecular Biology (ISMB) 2009 and heard Daphne Koller’s keynote. What a shocker—point by point, I could tick things off of my to-do list. Not only had Daphne’s lab thought of all my ideas for module networks, but they had implemented, tested, and improved them, and the papers had already begun to be published [2]. Well, I thought, at least they are not doing this in the field of cancer. But then I saw one of Dana Pe’er’s publications [3], which killed my research program for good. I could have added some marginal improvements, sure, but that wouldn’t have been too exciting. So more than a year into my Principal Investigator (PI) position, I stood there empty-handed, without much of an idea what to do next. I had hit the ground, but I wasn’t running. What rescued me was the people in my group.

## Balancing Leading with Listening

One of my first postdocs came with a background in phylogenetics, and he wanted to do an evolutionary project in cancer. At first, I resisted because I didn’t know anything about evolution, and the whole idea didn’t seem to fit well with my research agenda of genomic data integration and machine learning. But when I saw how motivated he was, I connected him with a neighbouring lab in our institute that was working on genetic heterogeneity in ovarian cancer. Over the next few years, projects on tumour heterogeneity and evolution became one of the most important research areas in my lab, which led to a string of papers [4–8].

Another major research project began when an undergraduate intern visited the lab for four months in 2009. When I proposed potential projects to him, the intern invariably gave me a Bartleby response: “I would prefer not to," before adding, “I would rather do image analysis." Again, I did not know anything about images and it didn’t seem to fit my research agenda, but when I saw his enthusiasm, I decided to give it a try. The project grew from there. Today, using image analysis to put genomics into a tissue context is one of the major projects of the lab. It led to one of our most visible papers [9], was the basis of the PhD work of one of my students [10], and even inspired the research agenda of my first postdoc when she started her own lab at the Institute of Cancer Research (ICR) in London. This is quite an amazing outcome for a four-month internship!

Looking back, getting scooped gave me the freedom to explore new directions. The lesson I learned was to trust my people, be open to their ideas, and follow them in their projects.

## The Evolution of Our Scientific Mission

The interactions I have described shaped the scientific mission of my lab. As a starting PI, I did not have a very clear view of where I was going. With a strong background in machine learning and statistics, I had some general ideas about what type of methods I wanted to develop (“module networks for data integration”), but actually, any project with exciting new data seemed attractive to me. However, I quickly realised that, for a successful career, I needed a better agenda than being a “gun for hire.” The discussions I had with my team members and with collaborating PIs helped me to sharpen the focus of my lab. Shaping my scientific vision was a continuous iteration of leading and listening, discussing new ideas, and deciding which of them were worth pursuing further. This was a very important process because I was expected to have a clearly defined research vision when I came up for tenure after five years.

Today I describe my group’s work as the “*systems genetics of cancer*.” Using a mix of experimentation, computation, and theory, we aim to characterise the influence of genomic variation on patient phenotypes (like survival) as well as intermediate phenotypes like gene expression, transcription factor binding, and tissue architecture. This defines the general themes of the lab: We work on the evolution and function of cancer genomes, we put genomic information in a tissue context, and we develop computational methods of network inference and analysis. While the general themes are now defined, I still rely on ideas from my team about how to bring these abstract concepts to life.

## The Hitchhiker’s Guide to the FMlab

At the moment, I have ten people working with me; four of them started in the second half of 2014, so almost half of the lab members are new. When someone is new to the lab, I want them to have a smooth and productive start, without much confusion about the goals of their particular project. This is why, for every new member, I generally prepare a two-page document that contains a short “elevator pitch” summary of the project goals, a reading list, and some milestones for the first few weeks, as well as a list of people to talk to. In my experience, a short document like this helps new team members to quickly settle down, become productive, and start shaping the project with their own ideas.

In addition to these project-specific two-pagers, I have described our lab culture in a longer, more general document called “*The Hitchhiker’s Guide to the FMlab*.” For example, the first section of the guide is called “You lead projects,” and it starts with the sentence “You don’t work *for* Florian. Florian works *with you* on your projects.” It continues, providing advice on how to choose and organise projects. Another part of the guide is called “You manage upwards,” and is based on the short article, “The care and maintenance of your adviser” [11]. The key sentence is, “Maintaining your adviser means asking for what you need rather than hoping that he will know what to provide.” My message to my team is that I am happy to support them in any way I can, but I expect them to come up with ideas and a plan.

The guide has grown over the years and is now 13 pages long—quite a lot to digest! This is why I don’t just hand out these pages (“Here’s some bedtime reading”), but use them as the basis for a discussion that I have with every new member after four weeks in the lab. In this conversation, I explain not only how we do things but also *why* we do them the way we do.

The reason I have put all this effort into writing these things down and discussing them with everybody is that I wanted to be explicit about my expectations. I believe that being transparent and predictable helps prevent frustration and conflict. Over the years, the new members in my lab have all reacted positively to the guide. They were happy that I had made explicit the rules that might, in other labs, be unwritten.

## This Is Not a Computer Science Department

Another expectation I often need to make clear is about the type of research we are interested in. My lab is part of an institute funded by Cancer Research UK, the world’s largest independent charity for cancer research. Yet most members of my team do not have a background in cancer biology; rather, they have been trained in computer science, engineering, or statistics. One of the first issues we need to discuss when new members join is how the biological research in a cancer research institute differs from the methodological research they might have been used to in a computer science (CS) or statistics department. Purely methodological research aims for general solutions and is often separated from the biological question that motivated the generation of the data in the first place. For someone in a CS or statistics department, it might be completely OK to be inspired by some biological problem and then go off to do their theoretical work without ever thinking of the biology again.

However, as I tell my new members, that’s not how it works for us! We are computational *biologists*. Our work is not only motivated by biological problems but it also actively tries to solve them by generating testable and tested results. The message is that if you work in my group, you need to start with an important biological problem and see the project through all the way to experimental validation. In particular, this means that I expect all of my team members to go to biological talks that are outside of their comfort zone, regardless of whether they initially think the talks are boring. I want them to develop a feeling for the biological and medical context in which we do our work.

My team members profit from the explicitness of my expectations because it helps to create an open and transparent environment that is supportive and empowering. My goal is to provide all students and postdocs with the wide range of skills they need for the next steps in their careers. This includes technical skills in data analysis but, even more importantly, it also involves coaching in how to identify profound questions and plan successful projects. I actively work with them on building a network of collaborators and increasing their visibility by giving presentations at conferences. As a result, my lab takes in specialised computer scientists, engineers, or molecular biologists, and helps to develop them into well-rounded cancer researchers. Of the five people (four postdocs, one PhD student), who have left my lab so far, all are pursuing a scientific career and four are now in faculty positions.

## Less Talking, More Listening

Communication is imperative for a successful lab. It took me a couple of years to establish an open, collaborative atmosphere. Here are some of the strategies I used.

### 2+1 meetings

In the beginning, when I had four people working with me, the communication in the lab looked like a star topology. Everybody seemed happy to talk to me, but no two people talked to each other. I thought this was weird because I could spot obvious overlap between the projects. Somehow, the weekly group meetings, in which everybody presented their newest results, were not enough to bring the group together. As a countermeasure, I developed what I call “2+1 meetings.” Once a week, randomly selected pairs of team members would meet with me, regardless of whether or not their projects seemed closely related. First, one person would discuss their project, then the other would talk. In each discussion, I actively tried to involve the other team member. Over time, the strategy succeeded and the team grew together more closely. Everyone knew much more about each other’s projects, and the overlap I envisaged from the beginning became apparent to everyone.

### Weekly group meetings

Like most other labs, we also have regular group meetings in which everybody comes together. One or two people give a talk, and we are pretty flexible with the format; sometimes it is just an update on recent results, sometimes it is the rehearsal for a presentation at a conference, and sometimes it is a “chalk talk” (without any slides) to brainstorm some ideas. At the end of every group meeting, we do a quick round of project updates from everybody and open the floor for any other business. This way, everybody feels integrated in the group meetings, even when they are not giving a talk.

### Strategy meetings

Every six months I have a strategic meeting with every team member. This is not about the details of their projects but rather about a bird’s-eye view of where they are going. Most computational researchers I know (and not only those in my group) seem to be flooded with projects, so it is important to develop prioritisation strategies. In a strategy meeting, we first list all their projects and important sub-projects. We then rank them all using three categories: 1) How far from completion is it? (“0” stands for “Here is some crazy idea I just had” and “100” for “publication ready.”) 2) How important is this project for the team member? That is, how important is it for their career and their personal research agenda? Here, I explicitly ask for their priorities, which are not necessarily my own. And finally, 3) how urgent is each project? Are there external deadlines? Is there pressure from collaboration partners? This is where my own priorities come in: I cannot easily make a project more important for them, but I can make it more urgent. Comparing the scores in these categories generally makes it very easy to decide where to put the focus in the next six months.

### Less talking, more listening

An important lesson I’ve learned in team meetings over the years is to listen more often than I talk. In the beginning, when I explained an idea and didn’t seem to get the response I wanted, I just continued to talk and repeat myself. I usually ended up standing at the white board while my team sat on the couch waiting for the lecture to be over. What I learned is that I should wait and let the idea settle. Quite often, after some time, people would explain back to me (sometimes even as their own ideas) what I thought I had failed to explain in the beginning. That’s when I knew I had actually been successful.

## A Project Is More than a Beautiful Result

### Thinking from the end

When we discuss projects, I always encourage team members to start with a vision of what the “perfect paper” would look like. What will be novel? What will be the conceptual advance? And how will we validate it? After establishing a big goal, we work our way backwards to the current stage of the project and develop a battle plan for how to proceed. In my experience, defining the ideal paper first and thinking backwards helps to avoid frustration later in a project. By thinking through all steps of a project early on, I avoid people thinking, “I have done so much already, my result looks so beautiful, and now my boss asks me to do yet another validation.” Instead, the validations (and many other tedious steps) are part of the plan from the beginning.

### Maintaining reproducibility

Over the years, I have had some bad experiences with reproducibility. In one case, our experimental collaboration partners spent almost two years validating a pathway model we generated. We all were very pleased with the outcome, and even started to throw around the names of glamorous journals to which we might submit our paper. There was only one tiny problem: no matter how hard we tried, we could not computationally reproduce our initial pathway model. We had many hypotheses about what had gone wrong; the data might have changed, or maybe the code, or maybe we just couldn’t remember the parameter settings correctly. We couldn’t figure out how it had happened, and, at the end of the day, it didn’t matter, the result was just the same: without a reproducible model, the project was dead.

I tell this story to new members early—even before they have written the first line of code or completed their first experiment—because I want them to be aware of the importance of reproducibility from the beginning. If I wait too long to explain reproducibility, they might think I am delaying their project and preventing them from publishing their beautiful (but potentially non-reproducible) result. For example, writing an R markdown document to reproduce all numbers and figures in the manuscript is not “yet another boring step holding up submission,” but an integral part of the project. It took some years to establish a culture of reproducibility in the lab, but now we version-control all data and all code, and we include well-documented scripts (mostly as R markdowns) as supplementary material to every manuscript we submit. Now that this culture is established, it is easy to maintain because new members see that everybody is already doing it. Aiming for full transparency and reproducibility is nothing special in my lab.

## Pitfalls to Avoid

### Hiring the wrong people

In the first few months after I had started my lab, one of my senior colleagues shouted, “Florian, don’t panic!” every single time he saw me in the corridor. I must have looked like a headless chicken while attempting to organise the lab. For example, I almost hired a biophysicist who was working on spatial models of DNA structure. He was not really interested in my stuff, and I was not interested in his; there was no common ground on which to build a joint project. But still, I got very close to hiring him—mainly because I felt lonely. Everybody had told me to “hit the ground running,” so I felt the pressure to fill all of my positions as quickly as possible. But that would have been a mistake! No matter what pressure you feel, wait until the right candidates turn up. Eventually, they will.

### Getting side-tracked

People like us, bioinformaticians and computational biologists, are terribly useful people to have around, and most of us find it very easy to start collaborations. But don’t forget about your own research agenda. Are you useful for someone else, or are you a leader yourself? Do you want a publication list full of middle-authorships, or do you want a recognisable research agenda? No matter how good an idea is, it takes a lot of hard work to develop, implement, and polish it. So it’s important to be very selective about which projects to take on and with which people to collaborate.

### Underestimating people problems

“Great minds discuss ideas. Small minds discuss people.” I have heard this quote often, and it might make sense occasionally. But science is already excessively cerebral and populated by terribly ambitious people full of “Big Ideas.” Here, it is toxic to believe that we “great minds” shouldn’t discuss people. Especially because all the important stuff you do as a group leader is about people. If you have achieved a PI position, it is safe to assume you are very good at doing science and really enjoy the work—and then the only remaining problems you will face are people-problems. How do you keep your team motivated if the results don’t look pretty? What do you do about conflict in the lab? How do you keep the lab atmosphere collaborative and nurturing? I never had to deal with these questions as a student or postdoc. It took me a while to realise that, in my new job as a group leader, people-problems were actually the difficult nuts to crack. The “real” science was comparatively easy—after all, I had completed years of training to do it.

## Final Thoughts for Future Leaders

I cannot tell you how to lead your group—too much depends on your personal style and character—but I can give some pointers about what skills you will need to acquire and how to go about it.

### Start thinking about leadership styles early

As a student or postdoc, read Duncan Odom’s papers [12,13] and start thinking about the impact of different leadership styles. Learn from the examples that your supervisors and other PIs set. Reflecting on how your own advisors deal with people issues in the lab, how they motivate their teams, and how they address conflicts will increase your leadership skills and benefit you throughout your career.

### Get leadership training

As a senior postdoc or fresh PI, take the European Molecular Biology Organisation (EMBO) lab management courses [14]. They are worth every penny! For most of us, these courses are the only training we will ever get on how to lead a lab. Science Careers recently produced an overview of many similar courses worldwide [15]. And for self-study, read a book like Landsberg’s *The Tao of Coaching* [16]. Despite the wacky title, this short book is a gem.

### Establish a support group

If you are lucky, you will find a peer support group at your institute. For example, the Weizmann Institute seems to be very successful with their Young PI forum [17], and their webpage has many resources [18]. If you are not lucky enough to find one, maybe start a Young PI group yourself. You will definitely need mentors you can turn to for feedback and advice. Ideally, you can establish good personal connections with someone who is a few years ahead of you (and can still relate to the daily struggles of a newbie PI) and someone much more senior who can guide you with their long-term vision.

### Widen your network

One of the most successful things I did in my first years as a new group leader was to organise a meeting at my university. I wanted to get to know more people in my community (especially since I had just moved back from the United States to Europe), and the easiest way was to get them all together for a meeting. I teamed up with a more senior colleague who already had a big network, and received some funding from the European Science Foundation to organise what they called an “exploratory workshop”—a small meeting of about 30 PIs from all over Europe. Participation was by invitation only, and the talks were very short and focussed, in order to leave room for discussion. The setup was so successful that I’ve kept doing it [19–22]. The talks are now all “chalk talks” (no PowerPoint slides at all!) to make sure they are conceptual and interactive. This setup is so different from other meetings that we never have a problem enticing the thought-leaders in a field, even though we do not pay travel expenses.

Locally, in my institute, I initiated a lecture series on computational biology. The institute supported me with some funds, and I started to invite speakers from all over Europe. This was really a great way to get to know people. In the beginning, these lectures were called “Lectures in Computational Biology,” and almost no one came; the word “computational” must have been too frightening. I renamed the series as “Lectures in Computational Biology *and Genomics*,” which attracted a bigger crowd, but ultimately I decided to call it “Lectures in Quantitative Biology,” which gave it a further boost. Now we have an audience of wet-lab and dry-lab researchers from all over town. I learned that “quantitative” is a much less frightening word than “computational.”

### There are many ways to lead a successful lab

What I have described here are the strategies that worked well (and still work) for me. If you succeed in building a communicative and collaborative environment in your group by balancing leading with listening, you will avoid the dangers of micromanaging your team (too much leading) and indecisiveness (too much listening). You might have to take a different road there than I did because the way you work with your team will depend on your own character and style. But I hope that the examples I have given will be helpful in choosing strategies that will work for you.
